# Carotenoids, Fatty Acid Composition and Heat Stability of Supercritical Carbon Dioxide-Extracted-Oleoresins

**DOI:** 10.3390/ijms13044233

**Published:** 2012-03-30

**Authors:** Cristiano Longo, Lucia Leo, Antonella Leone

**Affiliations:** Unit of Lecce (ISPA-CNR), Institute of Sciences of Food Production, National Research Council, Via Prov.le Lecce-Monteroni, 73100 Lecce, Italy; E-Mails: cristianolongo@libero.it (C.L.); lucia.leo67@yahoo.it (L.L.)

**Keywords:** tomato oleoresins, *cis-*, *trans*-lycopene, heat stability, antioxidant activity, hazelnut, supercritical-CO_2_ extraction, nutraceuticals

## Abstract

The risk of chronic diseases has been shown to be inversely related to tomato intake and the lycopene levels in serum and tissue. *Cis*-isomers represent approximately 50%–80% of serum lycopene, while dietary lycopene maintains the isomeric ratio present in the plant sources with about 95% of all-*trans*-lycopene. Supercritical CO_2_ extraction (S-CO_2_) has been extensively developed to extract lycopene from tomato and tomato processing wastes, for food or pharmaceutical industries, also by using additional plant sources as co-matrices. We compared two S-CO_2_-extracted oleoresins (from tomato and tomato/hazelnut matrices), which showed an oil-solid bi-phasic appearance, a higher *cis*-lycopene content, and enhanced antioxidant ability compared with the traditional solvent extracts. Heat-treating, in the range of 60–100 °C, led to changes in the lycopene isomeric composition and to enhanced antioxidant activity in both types of oleoresins. The greater stability has been related to peculiar lycopene isomer composition and to the lipid environment. The results indicate these oleoresins are a good source of potentially healthful lycopene.

## 1. Introduction

Lycopene is the most represented carotenoid in tomato, accounting for above 90% of the total carotenoids and it is one of the major carotenoids in the Western diet. Lycopene, an acyclic isomer of β-carotene, without provitamin-A activity, is a natural red pigment synthesized by plants and microorganisms but not by animals. It is a highly unsaturated, straight chain hydrocarbon containing 11 conjugated and two non-conjugated double bonds. Serum and tissue lycopene levels have been inversely related with the risk of chronic diseases [1,2], providing explanation for the decreased risk of several pathologies, including cancer and cardiovascular disorders, correlated with a dietary intake of tomatoes and tomato-based foods [3,4]. Although interest in lycopene was firstly focused on its antioxidant properties, their beneficial effects seem to be related also to other mechanisms such as modulation of intercellular gap junction communication [5–7], hormonal and immune system, and inflammation response [8–10].

Naturally occurring geometrical isomers of lycopene are primarily in an all-*trans* configuration with few exceptions. Dietary studies showed that the most available sources of ingested lycopene maintain the natural isomeric distribution ratio with about 95% in the all-*trans* form. However *cis*-isomers of lycopene represent approximately 50% of total lycopene in blood and up to 80% in prostate tissues [10]. This suggested that *cis*-isomers are more bioavailable than all-*trans*-lycopene, most likely because of the greater solubility of *cis*-isomers in the bile acid micelles, a shorter chain length to fit into micelles, and the lower tendency to aggregate [11,12]. Recently an “in cell” isomerization has also been hypothesized [13]. The mechanisms explaining the isomerization of all-*trans* to *cis*-lycopene isomers *in vivo*, and the physiological importance of *cis*-lycopene are not fully understood. During food processing, lycopene may isomerize to *cis*-isoforms with the presence of heat and/or oil, or during dehydration. Moreover, during storage and/or processing lycopene undergoes further geometrical isomerization, mainly *cis-* to *trans* retro-isomerisation [14 and citations therein]. With long heating times or temperatures above 50 °C, degradation proceeds faster than isomerization, the stability of lycopene isomers decreases in the order: 5-*cis* > all-*trans* > 9*-cis* > 13*-cis* > 15*-cis* > 7*-cis* > 11-*cis*. Starting from the all-*trans*-lycopene, isomerization is characterized first by the formation and then by the disappearance of the unstable 13-*cis*-isomer [14,15]. It is clear that isomerization and degradation are competitive and contemporaneous processes.

Therefore, tomato extracts with a stable lycopene isomer profile, which do not undergo retro-isomerization, containing no unstable *cis*-isomers, would be an ideal source of highly bioavailable lycopene, greatly demanded for foods and nutritional supplements. Tomato oleoresins enriched in lycopene (a mixture of flavor compounds, pigments, fats, fatty acids, and sterols usually extracted by organic solvents) are readily absorbed and act as an *in vivo* antioxidant, in addition they are used in foods and supplements to enhance the nutritional value, functionality, color, and flavor. Lycopene is highly requested not only by food companies but also for the feed, cosmetic and pharmaceutical industries. For these reasons there is a great interest in the use of environmentally friendly processes for industrial production of lycopene-containing oleoresins, able to preserve the biologic properties of the components and free of residues of potentially toxic organic solvents. As a consequence many efforts are continuously made to improve extraction methodologies in order to obtain oleoresin enriched in lycopene, possibly having an isomeric composition valuable for its stability and bioavailability, and to avoid the toxic effects of the conventionally used organic solvents.

New extraction methods able to overcome the drawbacks of the traditional methods are being studied; among them the Supercritical Fluid Extraction (SFE) is the more promising process [16]. SFE provides higher selectivity, shorter extraction time and do not use toxic organic solvents. Supercritical CO_2_ extraction (S-CO_2_) has been used to extract lipid compound from numerous plant matrices [17] and has been extensively developed in recent years to extract lycopene from tomato and tomato by-products [18,19]. Many studies are performed on the S-CO_2_ extraction of lycopene, focusing on technical parameter in order to increase the rate and yield of extraction and to preserve the antioxidant activity also by adding safe co-solvents such as ethanol and vegetal oils [18,20]. Beside the use of co-solvents, the modification of the starting matrix, by adding extra-matrices useful to improve the extraction yield and the quality of the final products, may be another successful strategy. This method, however, resulted in the passage of some allergenic hazelnut proteins in the oleoresin, during the extraction [21]. In our previous studies oleoresins with higher antioxidant activity and peculiar biological properties on human cell cultures were obtained by using hazelnut [7] and grape seed [5] powders, as co-matrices.

Increasing requirements from both consumers and producers, for a better understanding of nutritional components in foods or supplements and the industrial processing influences on them, are resulting in researches focused to the analysis of biochemical features and stability of S-CO_2_ extracted oleoresins. Few studies have been so far reported on the thermal stability and isomerization of lycopene in tomato oleoresins after the S-CO_2_ extraction process. The aim of this study was to elucidate the physicochemical features of tomato oleoresins obtained from tomato matrices added, or not, with hazelnut powder, used as co-matrix, in S-CO_2_ extraction process. The carotenoid composition, the thermal stability and isomerization of lycopene and its antioxidant activity in such tomato oleoresins, were investigated.

## 2. Experimental Section

### 2.1. Reagents and Standards

Methanol, ethanol, hexane, ethyl acetate, acetonitrile (HPLC grade) and acetic acid were purchased from Merck Darmstadt, Germany; lutein, β-carotene, *trans*-lycopene, potassium persulfate (dipotassium peroxdisulfate), 6-hydroxy-2,5,7,8-tetramethylchroman-2-carboxylic acid (Trolox) was purchased from Hoffman-La Roche. 2,20-azinobis (3-ethylben-zothiazoline-6-sulfonic acid) diammonium salt (ABTS) and FAME mix were purchased from Sigma-Aldrich.

### 2.2. Plant Material, Matrices and Lycopene Extraction

Ripe fresh tomatoes (*Solanum lycopersicum* L. var. *ramato*) grown in Apulia were purchased from a local market. Tomatoes were crushed and autoclaved for 20 min at 120 °C, then the pulp was separated from the seeds and skins, and centrifuged at 4400× g (Bechman, J2-21) for 30 min, at 4 °C. The resulting tomato paste was dried in a vacuum drying oven (Salvis Lab, IC40) at 40 °C and the obtained dried powder (about 18 mesh), was stored under vacuum, at −20 °C, protected from light. Hazelnut (*Corylus avellana* L.) powder was obtained from commercial toasted hazelnut crushed by a domestic blender and immediately used as extraction matrix. Tomato powder and tomato added with hazelnut powder, in a ratio 1:1 (w/w), obtained at the CNR-ISPA lab, were used as extraction matrices in S-CO_2_ extraction.

#### 2.2.1. S-CO_2_ Extractions

The extractions with S-CO_2_ were performed in collaboration with the company Pierre s.r.l., (Galatina, Lecce, Italy) as described in [18]. Briefly, a pilot plant equipped with an extractor maintained at a temperature between 60–70 °C and properly pressurized carbon dioxide, from 400 to 450 until the final value of 30–40 bar, were used. The extractions were run by submitting 2.0 kg of the tomato powder or tomato powder/ hazelnut mixture in a 1:1 (w/w) ratio, prepared as above described, to a pressure of 400–450 bar and a temperature of 60–70 °C. The flow rate of CO_2_ was in the range of 18–20 kg CO_2_/h and the extraction time was about 7 h. All the extracted oleoresins, from each matrix type, was pooled and stored at −20 °C.

#### 2.2.2. Solvent Extraction

The tomato powder was suspended in tetrahydrofuran (THF) in the ratio 1:100 (w/v), the sample was saturated with gaseous nitrogen and the extraction was performed at room temperature, for 20 min, under stirring in quick fit, and under dim light. The extract was centrifuged at 4300× g (Hereus, Megafuge 1.0) for 10 min, at room temperature. The surnatant was used for the analyses.

The solvent extract and the two lycopene-enriched oleoresins obtained by S-CO_2_ extraction from tomato matrix (S-CO_2_ExT) and tomato plus hazelnut mixtures (S-CO_2_ExTH) were used for qualitative and quantitative analysis of carotenoids, *in vitro* total antioxidant activity and evaluation of their heat stability. All below described procedures were performed by replacing air with gaseous N_2_ and under dim light to reduce oxidation processes.

### 2.3. Treatments of Supercritical CO_2_-Extracted Oleoresins

Oleoresins extracted by S-CO_2_ (1 mL) were centrifuged at 9000× g (Beckman, Allegra 21R) for 15 min at 20 °C. An oily fraction (supernatant) was separated from an insoluble paracrystalline fraction (pellet). The two fractions were weighed and characterized for qualitative and quantitative analysis of carotenoids and for the *in vitro* antioxidant activity.

For the evaluation of the oleoresins stability to high temperatures, the whole oleoresins were kept at 25 °C, 60 °C, 80 °C and 100 °C for 15 min in a water bath. After the treatments, the oleoresins were centrifuged at 9000× g for 15 min (Beckman, Allegra 21R) at room temperature. The oil-fraction and the paracrystalline fraction were separated, weighed and then characterized for qualitative and quantitative analysis of carotenoids and for the *in vitro* antioxidant activity as described below.

### 2.4. Qualitative and Quantitative Carotenoids Analysis

Carotenoids were separated by stirring 0.1 g of each of the two S-CO_2_-extracted oleoresins in 10 mL of hexane/acetone/ethanol (2:1:1, v/v/v) solution, and then an equal volume of distilled water was added, followed by 5 min of stirring. The same solvent mixture, and 1:100 (w/v) sample/solvent ratio were used to solubilize the oil- and the insoluble fractions of the not-treated and heat-treated oleoresins. After separation of the aqueous phase from the hexane, 20 μL of the hexane solution containing carotenoids were injected into the high performance liquid chromatography (HPLC) system 126 solvent module equipped with a System Gold 168 diode array detector (Beckman-Coulter, Fullerton, CA). The analysis was carried out using a reverse phase (RP) C30 column (4.6 mm 2.5 cm, YMC, Milford, MA). A methanol/methyl butyl ether/ethyl acetate (50:40:10) solution was used as mobile phase, at a flow rate of 1 mL/min. Carotenoid detection was achieved at a 475 nm wavelength. Chromatographic peaks were identified by the addition of internal standards or by comparison of the absorption spectra of unknown peaks with reference standards. Carotenoid quantification was achieved by comparison with the standard curves of lutein, β-carotene, and *trans*-lycopene authentic standards (Sigma).

### 2.5. Lipid Determination

Total lipids were extracted from S-CO_2_ oleoresins as described in [5]. Briefly, 0.05 g of the oleoresins were stirred in 5 mL of hexane/methanol solution (2:1, v/v), followed by the addition of an equal volume of water. The two phases were separated by centrifugation, and the upper lipid-containing phase was concentrated on a rotary evaporator under vacuum. Total lipids were saponified with 2 M potassium hydroxide in methanol and re-extracted by hexane. The hexane layer was separated and analyzed by a gas chromatograph coupled to a mass spectrometer (QP5050 GCMS, Shimadzu, Kyoto, Japan) equipped with a 30 m DB-5MS (J&W Scientific, Folsom, CA) capillary column (0.25 mm i.d., 0.25 μm film thickness) for the determination of fatty acid methyl esters (FAMEs). The oven temperature profile was as follows: 80 °C for 5 min raised to 100 °C at 10 °C/min and then to 250 °C at 5 °C/min and maintained at this temperature for 15 min. The injector and interface temperatures were 250 °C. Helium was used as carrier gas at a flow rate of 1.1 mL/min. The fatty acids were fragmented by electron impact at 70 eV, identified by a comparison of their retention time and mass spectrum with those of standard FAME, and quantified according to their percentage area by integration of the peak.

### 2.6. *In Vitro* Antioxidant Activity Analysis

The total antioxidant activity was determined spectrophotometrically by using the Trolox Equivalent Antioxidant Capacity (TEAC) method, as described in [5]. The samples were solubilized in tetrahydrofuran (THF) in the ratio 1:100 (w/v) and then centrifuged at 4300× g (Hereus, Megafuge 1.0) for 10 min, at room temperature. A Trolox calibration curve in a range of 2.5–30 μM was prepared under the same conditions of the samples. The antioxidant capacity of the samples was calculated, on the basis of the inhibition exerted by standard Trolox concentrations at 734 nm, inhibition time being fixed at 6 min. Results were expressed as mmoles of Trolox equivalents per gram of oleoresin or per mg of contained lycopene.

### 2.7. Analytical Quality Control

In the HPLC separation of carotenoids, linear calibration curves were obtained for lutein (0.25–20 μg/mL, *R*^2^ = 0.9999), beta-carotene (0.625–50 μg/mL, *R*^2^ = 0.9997), lycopene (1.25–10 μg/mL, *R*^2^ = 0.9997). Repeatability of the HPLC method was estimated by running a standard solution containing each compound at decreasing concentration after repeated runs. After HPLC runs, the purity of analytes was checked by matching the UV/visible spectra of each peak with those of the standards. The limit of detection was calculated as the concentration yielding a signal-to-noise ratio of 2:1. Repeatability of the GC-MS in the fatty acids analysis was evaluated both for samples and standards. Samples were prepared for the instrumental analysis and injected 3 times. The standard FAME mix (Sigma Aldrich St. Louis, MO, USA) with known amounts of fatty acids: palmitic acid (16:0), palmitoleic acid (16:1), stearic acid (18:0), oleic acid (18:1) linoleic acid (18:2), linolenic acid (18:3), was analyzed in triplicate to test the recovery. The fatty acids were identified by comparison of their retention time and mass spectrum with those of standards, and quantified according to their percentage area by integration of the peaks. Precision of the TEAC results was evaluated by long-term variation (reproducibility) over a period of three months. This within laboratory reproducibility (day-to-day precision) was studied by conducting the whole method from sample extraction to spectrophotometry analysis in different weeks. The SDs were in the range 0.5–12% for TEAC. Quantification based on Trolox calibration curve, a good linearity with R^2^ values about 0.97 was obtained. Sample was extracted and analyzed each time in triplicate.

### 2.8. Statistical Analysis

When indicated, comparisons between mean values from control and treated samples were carried out with the two-tailed unpaired Student’s t test and a one-way analysis of variance (ANOVA). A value of *p* < 0.05 was considered to be significant.

## 3. Results and Discussion

### 3.1. Tomato Oleoresins

**Figure 1:**
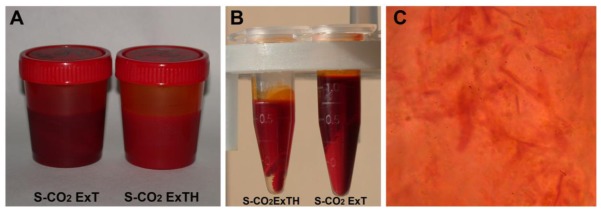
(**A**) Supercritical-CO_2_-extracted oleoresins obtained from pure tomato powder (S-CO_2_ExT) and from tomato powder added with hazelnut powder (S-CO_2_ExTH); (**B**) oil- and insoluble fractions after centrifugation; (**C**) light microscope image of the crystal-like material scattered into the oil phase in the S-CO_2_ extracted oleoresins (20×).

Figure 1 shows the two oleoresins obtained by S-CO_2_ extraction from either, pure tomato powder (S-CO_2_ExT) or from tomato powder added with an equal amount of dry hazelnut powder (S-CO_2_ExTH). Hazelnut powder was used as co-matrix, in order to improve the extractability of lycopene by S-CO_2_ methodology. The oleoresins obtained from the two plant matrices were compared for their quali-quantitative composition in carotenoids and other lipids, and for their heat stability and antioxidant activity. Both S-CO_2_-extracted oleoresins showed a characteristic strong red color and a high carotenoid content. The oleoresins exhibited a peculiar physical status where finely granular material appeared as crystal-like dispersed in an oil phase. The biphasic appearance was evident after centrifugation of the whole oleoresins (Figure 1).

### 3.2. Lycopene Content and Carotenoid Composition in Tomato Oleoresins

**Figure 2:**
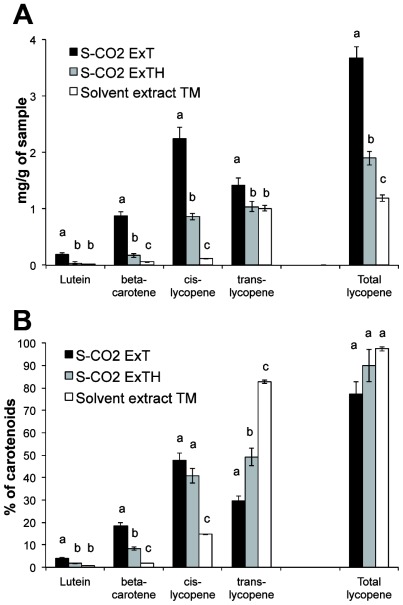
Carotenoid composition. Carotenoids and total lycopene content, expressed as mg per gram of oleoresin or tomato powder (**A**) or as percentage of the total carotenoids (**B**), in tomato powder extracted by hexane (Solvent extract), in the S-CO_2_ oleoresin from pure tomato powder (S-CO_2_ExT) and from tomato powder added with hazelnut powder (S-CO_2_ExTH). Data are mean ± SD of three measures from three independent samples.

Figure 2 shows the carotenoid composition detected in both the oleoresins obtained from tomato (S-CO_2_ExT) and tomato/hazelnut (S-CO_2_ExTH), compared with the carotenoids obtained by conventional hexane extraction from tomato powder (Solvent extract TM). The carotenoids detected in all the extracts were all-*trans*-lutein, β-carotene, as di-*cis*-β-carotene, *cis*-β-carotene and all-*trans*-β-carotene, and the lycopene isomers: 9-13-di-*cis-*, 13-*cis*-, 9-*cis*-, *cis*- and all-*trans*-lycopene, the last representing the main detected carotenoid (over 95% of the total carotenoids). In order to simplify the analysis, all the carotene isomers were referred as “β-carotene” and all the *cis*-isomers of lycopene were referred as “*cis*-lycopene”. The hexane extraction yields a quite complete extraction of lycopene from the tomato powder. The amount of extracted lycopene was 1.19 mg per gram of tomato powder, representing the 97.4% of the total extracted carotenoids (Figure 2A,B). The total lycopene detected in the oleoresins extracted by S-CO_2_ was 3.67 mg and 1.90 mg of lycopene per gram of tomato powder in S-CO_2_ExT and S-CO_2_ExTH, respectively. Thus, the lycopene concentration in both the S-CO_2_-oleoresins was higher compared to the starting tomato powder. In the S-CO_2_ExT the total lycopene content was two times higher than in the S-CO_2_ExTH, as expected for the dilution effect caused by the hazelnut addition to the matrix.

Substantial differences, only quantitative, in the carotenoid composition were observed among the different extracts. Lutein, β-carotene and lycopene were higher in the S-CO_2_ExT oleoresin, compared to S-CO_2_ExTH and solvent extract. A significant difference was detected in the *cis*-/*trans*-isomers of lycopene. The solvent extract contained mainly all-*trans*-lycopene (*cis*-lycopene was 9.92% of the total carotenoids) and the *cis*/*trans* ratio was 0.18, reflecting the ratio in the tomato powder, and very similar to the ratio in the tomato fresh fruit (0.16, data not shown). Inversely, the amount of *cis*-lycopene was higher in the S-CO_2_ extracted oleoresins compared to the solvent extract, being about 47.7% and 40.7% in the S-CO_2_ExT and S-CO_2_ExTH, respectively. In addition the *cis*/*trans* ratio was inverted in the two types of oleoresins, being 1.60 and 0.83, in S-CO_2_ExT and S-CO_2_ExTH, respectively. This was likely due to the different rate of isomerisation of the lycopene during the supercritical fluid extraction and/or to a selective *cis*-isomer extraction of lycopene, possibly depending from its solubility in the different extraction environment [22].

**Figure 3:**
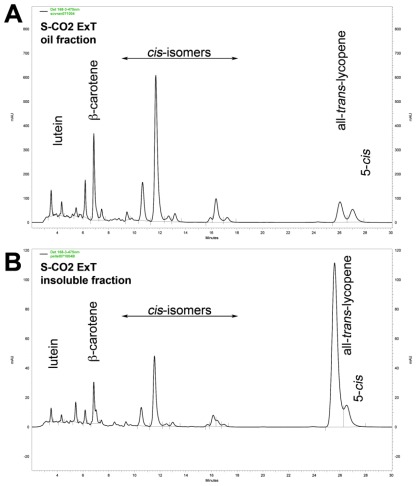

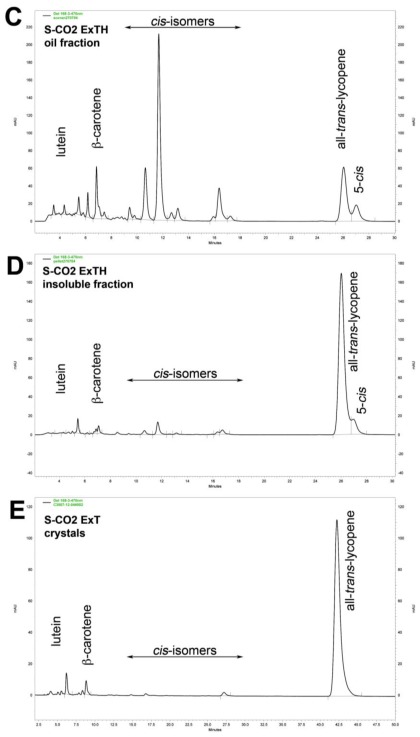
Chromatographic profiles of the high performance liquid chromatography (HPLC) analysis of carotenoids. Oil-fraction (**A**) and insoluble-fraction (**B**) of the oleoresin S-CO_2_ ExT extracted from pure tomato powder. Oil-fraction (**C**) and insoluble-fraction (**D**) of the oleoresin S-CO_2_ ExTH extracted from tomato/hazelnut powder. Insoluble paracrystalline fraction of S-CO_2_ ExT oleoresin after re-precipitation in hexane (**E**).

The analysis of the carotenoids was extended also to the separated oil- and insoluble-fractions of both the oleoresins, providing different HPLC profiles as showed in the Figure 3. Interestingly, it was evident a different isomeric distribution in the two fractions of both the oleoresins, where the all-*trans*-lycopene was mostly present in the insoluble fractions (Figure 3B,D) and the *cis*-isoforms were predominantly in the oil fractions (Figure 3A,C). When the insoluble material of both oleoresins, were solubilized once in hexane and stored at low temperature (−20 °C, 16 h), a re-precipitation of a crystalline material consisting of almost pure all-*trans*-lycopene (Figure 3E), was observed. It can be emphasized that this appeared to be an easy method to purify all-*trans*-lycopene from this kind of complex oleoresins. The 15-*cis*-, 13-*cis*-, 9-*cis*-, 5-*cis*-lycopene and a number of di-*cis*-isomers have been identified in both fractions of the oleoresin S-CO_2_ExT and in the oil-fraction of the oleoresin S-CO_2_ExTH.

Table 1 shows the quantitative carotenoid composition detected in the oil- and insoluble fractions of the two oleoresins obtained from tomato (S-CO_2_ExT) or tomato added with hazelnut (S-CO_2_ExTH). The total lycopene content, as *cis* and *trans* isoforms, in the oil fraction and insoluble fraction of the S-CO_2_ExT oleoresin, was 306 mg/100 g and 582 mg/100 g, respectively. In the oleoresin S-CO_2_ExTH the total lycopene was 118 mg/100 g and 306 mg/100 g in the oil- and insoluble fraction, respectively. The amount of total lycopene present in tomato matrix extractable by hexane was 119 mg/100 g of tomato powder. Consequently, the lycopene concentration increased up to five times in the S-CO_2_ extracted oleoresins. Quantitative analyses have confirmed that the insoluble-fractions were enriched in lycopene compared to the oil fractions. Again, the *cis/trans*-lycopene ratio was very different in the two fractions: the more stable trans-lycopene was mostly in the insoluble fractions, whereas the oil fractions contained higher amounts of *cis*-lycopene. The all-*trans*-lycopene, in the insoluble fraction of the oleoresins S-CO_2_ExT and S-CO_2_ExTH, was 66% and 83%, respectively, while in the corresponding oil fractions was 17% and 29% of the carotenoids, respectively. These results have established that the different tomato S-CO_2_-extracted oleoresins have a complex carotenoid composition related to the physical status, with particular reference to isomeric composition of lycopene. These products may provide lycopene sources, enriched in one or in the other isomer, easily separable. The food market may also be interested in a product rich in *cis*-lycopene, since many studies have suggested that *cis*-isomers, such as 15-*cis*, 13-*cis*-, 9-*cis*- and 5-*cis*-lycopene, are present in human serum and seem more bioavailable [23–25].

### 3.3. Fatty Acid Composition in Tomato Oleoresins

The lipids present in the oleoresins were essentially triglycerides, diglycerides and monoglycerides (data not shown). Table 2 shows the lipid fatty acid percentage composition in the two oleoresins and in tomato matrix. Saturated fatty acids (FAs) as palmitic and stearic acids, monounsaturated FA as oleic acid, and polyunsaturated fatty acids (PUFA) as linoleic and linolenic acids, were detected in both oleoresins and tomato matrix. However, S-CO_2_-extraction results in enrichment in oleic acid, which represented above 80% of the total lipids in the oleoresins. Traces of palmitoleic acid (FA derived from hazelnut) were present only in the S-CO_2_ExTH. Only small amounts of linoleic and linolenic acids were detected in S-CO_2_ extracted oleoresins, likely due to the susceptibility to oxidation of these PUFAs during extraction and/or storage. The lipid environment (including lipids present in the tomato matrix and vegetal co-matrix) is certainly of high importance during the extraction, affecting on the lycopene yield and on lycopene isomer composition and stability [26]. Moreover, lipids also affected the isomeric composition of lycopene, due to the different lycopene solubility in oil. In addition, lipid environment during extraction and storage may have a dual effect: lycopene protection from oxidation or, conversely, lycopene depletion triggered from the oxidized products of several easily oxidized lipids. The hazelnut-containing matrix has yielded an oleoresin with a lower lycopene concentration, but the total amount of oleoresin obtained from the same amount of matrix was notably higher (data not shown). The direct addition of the hazelnut powder was an easier and cheaper method to manage the tomato matrices. However, this method resulted in the removal of some hazelnut proteins from the hazelnut matrix and in their dragging in the oleoresin during the extraction. The extraction of proteins, their SDS-PAGE and Mass Spectrometry analyses have indicated the presence of highly allergenic protein coming from hazelnut in the S-CO_2_-extracted oleoresins [21].

### 3.4. Antioxidant Activity of Total and of Oil- and Insoluble-Fractions

**Figure 4:**
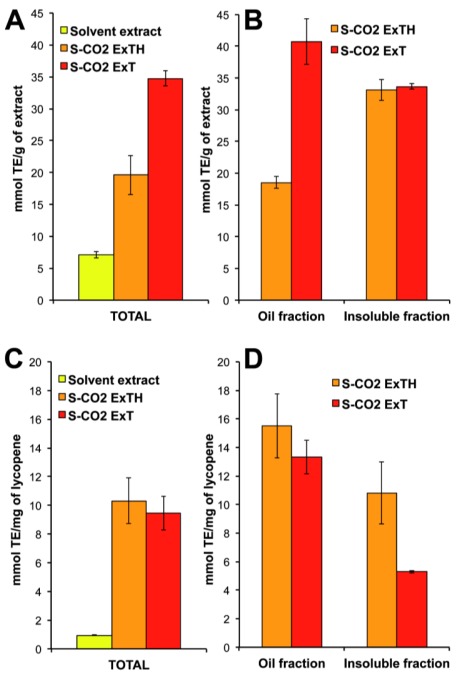
Antioxidant activity in the total extracts, oil- and insoluble-fractions of the S-CO_2_ extracted oleoresins. Antioxidant activity was expressed as mmol of trolox equivalent (TE) per gram of sample (**A**,**B**) or per mg of contained lycopene (**C**,**D**). S-CO_2_ExT: oleoresin extracted by S-CO_2_ from pure tomato powder; S-CO_2_ExTH: oleoresin extracted by S-CO_2_ from tomato/hazelnut powder; Solvent extract: tomato powder extracted by hexane. Data are mean ± SD of three measures from three independent samples.

Figure 4 shows the antioxidant activity measured in the two oleoresins, as total suspensions and as oil- and insoluble-fraction. Both the S-CO_2_-extracted oleoresins exhibited higher antioxidant activity, per gram of sample, compared to the hexane extract (Figure 4A). Antioxidant activity values were three times higher in the S-CO_2_ExTH (19.6 mmol TE/g), and five times higher in the S-CO_2_ExT (34.7 mmol TE/g) compared with the solvent extract (7.1 mmol TE/g). A large part of the antioxidant activity was in the insoluble fractions, which showed identical activity in both oleoresins, when expressed per gram of extract (about 33 mmol TE/g) (Figure 4B). The oil-fraction of the tomato oleoresin (S-CO_2_ExT), showed a significantly higher activity compared to the oil fraction of the oleoresin obtained from tomato/hazelnut matrix (40.7 mmol TE/g and 18.6 mmol TE/g, respectively). Since the major antioxidant component of oleoresin was lycopene, and because its concentration was different in the different samples, the values of antioxidant activity were expressed per mg of contained lycopene, as shown in Figure 4C,D. No significant differences between the antioxidant activities of the two S-CO_2_-extracted oleoresins, as total suspensions, were found (10.3 and 9.5 mmol TE/mg of lycopene, respectively). The values of antioxidant activity were really higher compared to that of the solvent extract of the tomato matrix (0.9 mmol TE/mg of lycopene) and to that measured in the standard all-*trans*-lycopene from Sigma (2.8 mmol TE/mg lycopene) in the same experimental conditions. Besides, the analysis on the separate oil- and insoluble-fractions showed that the lycopene in the oil fractions had higher antioxidant activity compared with the lycopene present in the insoluble fractions. As well, the difference between the oil-soluble lycopene in the two oleoresins was not significant (15.5 and 13.3 mmol TE/mg of lycopene, respectively). The major differences were detected in the insoluble fraction, where the antioxidant activity of lycopene in S-CO_2_ExTH was twice higher than in S-CO_2_ExT (10.8 and 5.3 μmol TE/mg lycopene, respectively). Likely, the antioxidant ability was mostly related to the amount of *cis*-isomers of lycopene. Indeed, *cis*-lycopene isoforms were predominant in the oil-fractions of both oleoresins (about 77% and 67% of total lycopene in S-CO_2_ExT and S-CO_2_ExTH, respectively) compared to the insoluble fractions (26% and 13%, respectively) as showed in Table 2. However, the differences in lycopene antioxidant performances seemed to be not merely determined from its chemical conformation. Indeed, the sample extracted by solvent, which contains *cis*-lycopene, showed yet a low antioxidant activity, similarly the all-*trans*-lycopene standard solubilized in hexane or THF showed very low antioxidant ability (2.8 μmol TE/mg of lycopene). It can be inferred that the difference in the lycopene antioxidant ability was not only due to its isomeric status but also to the physicochemical environment. In the oleoresins, the presence of other lipophilic antioxidants could stabilize and/or protect the contained lycopene. On the other hand, should be noted that certain lipids highly susceptible to oxidation, like PUFAs, can be oxidized forming highly reactive radicals, resulting in antioxidant depletion. In the oleoresin obtained from tomato/hazelnut matrix, for instance, the antioxidant tocopherols, highly concentrated in hazelnut [27], may exert a protective mechanism to prevent PUFA and lycopene from being peroxidised.

### 3.5. Effect of Temperature on Carotenoid Composition of Oleoresins

**Figure 5:**
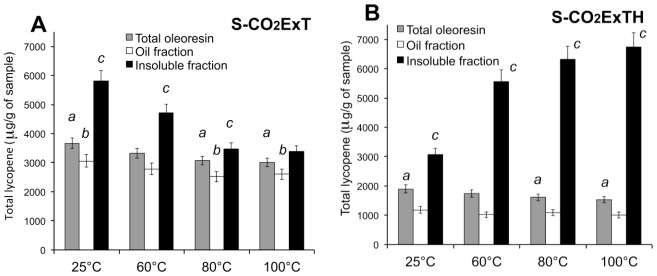
Effect of temperature on lycopene content in the oil and insoluble oleoresin fractions. (**A**) S-CO_2_ExT, oleoresin extracted from pure tomato powder; (**B**) S-CO_2_ExTH, oleoresin extracted from tomato/hazelnut powder. Data are mean ± SD of three measures from three independent samples.

The heat stability of oleoresins, and oleoresin fractions, were assessed by thermal treating of the two whole oleoresins, in the temperature range 25–100 °C. The isomeric composition and the antioxidant activity have been evaluated in both, oil- and insoluble fractions, separated after heat treatments. The total lycopene content detected in both the oleoresins, as whole (Figure 5A,B grey bars), did not seem influenced by the temperature at 60 °C, but the heating at 80 °C and 100 °C induced a decreasing of the detected total lycopene, as compared to non-heated (25 °C) samples. In the oil fractions (Figure 5A,B white bars) no changes (S-CO_2_ExTH) or a weak decrease (S-CO_2_ExT) at 80 °C and 100 °C in lycopene content was detected. Changes in lycopene content, instead, were detected in the insoluble fractions of both oleoresins. In S-CO_2_ExT, the detected lycopene resulted in a weakly but significant decrease when heated at 60 °C, 80 °C and 100 °C, as compared to not heated (25 °C) samples. (Figure 5A, dark bars). Conversely, a steady and significant increase of the total lycopene, at 60 °C, 80 °C and 100 °C, as compared to the sample kept at 25 °C, was evident in the insoluble fraction of the oleoresin ExCO_2_TH (Figure 5B, dark bars). In the oleoresin obtained from tomato/hazelnut matrix, the increase of lycopene content, which gradually occurs parallel with the increased of temperature, was likely due to the increased solubility of the insoluble crystalline-like lycopene. The oleoresin ExCO_2_T undergoes rather to a depletion of lycopene during oleoresin heating, likely due to a lycopene degradation caused of the absence of a protective lipidic environment.

**Figure 6:**
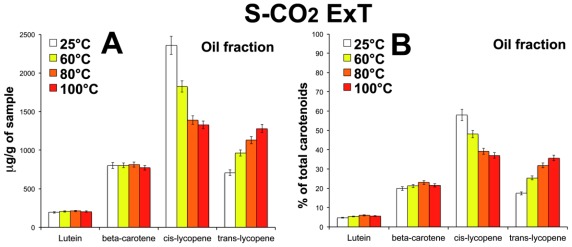

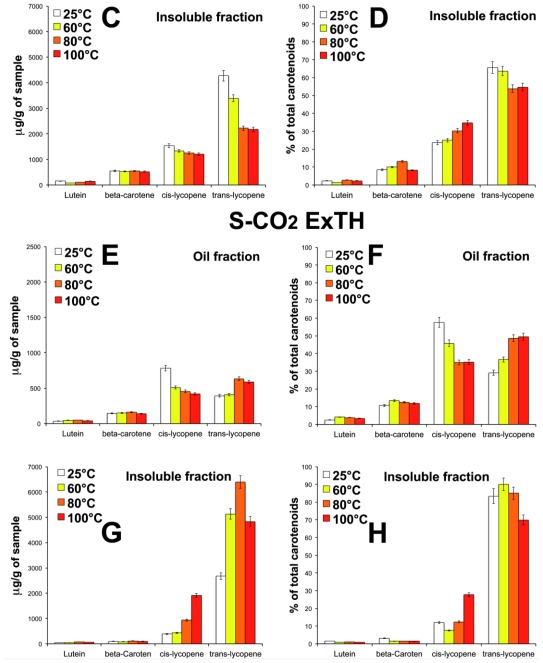
Effect of temperature on the carotenoid composition. Oil fractions (**A**,**B**,**E**,**F**) and insoluble fractions (**C**,**D**,**G**,**H**) of the oleoresin S-CO_2_ExT, extracted from pure tomato powder (**A**,**B**,**C**,**D**) and of the oleoresin S-CO_2_ExTH extracted from tomato/hazelnut powder (**E**,**F**,**G**,**H**). Values are expressed as mg per gram of sample (**A**,**C**,**E**,**G**) and as percentage of the total carotenoids (**B**,**D**,**F**,**H**). Data are mean ± SD of three measures from three independent samples (*n* = 9).

Lycopene is highly susceptible to oxidative degradation because of its highly conjugated polyenic structure. The *trans* forms of carotenoids are reported to be thermodynamically more stable than the *cis* forms. Anguelova and Warthesen [28] reported that during storage of tomato powder at 75–100 °C, several *cis*-isomers of lycopene were formed from all-*trans* lycopene. Temperature and time dependent isomerization of all-*trans* lycopene to *cis*-isoforms was reported in different tomato oleoresins [29]. *Cis*-lycopene isoforms in oleoresins would be more reactive compared with the oleoresins containing the more stable all-*trans*-lycopene. *Cis*-isoforms may undergo different degradation and isomerization mechanisms than the all-*trans* lycopene. All-*trans* lycopene was reported to be more stable in safflower oil or olive oil compared to oil-in-water emulsion [26, 30]. It was also showed that lycopene in oleoresin degrades predominantly through oxidation below 50 °C, and through isomerization at 75–100 °C [29]. In any case the autoxidation of either, the all-*trans* or *cis* isomer intermediates, was likely the major pathway for lycopene degradation. Thus, the different rate of lycopene degradation in different oleoresins may depend from the other components present in the oil phase of oleoresins, protective antioxidant (such as tocopherols) or high temperature generated free radicals of FAs, which can affect the lycopene degradation. Figure 6 shows the carotenoid composition, expressed as μg/g of sample and as percentage of the total carotenoids, of the two fractions of both oleoresins, subjected to 25 °C, 60 °C, 80 °C and 100 °C. Lutein, β-carotene and *cis-* and *trans-*lycopene, were always detected in all samples. In order to simplify the analysis, all the *cis*-isomers of lycopene were considered together. The results showed that heating treatments induced not significant changes in the lutein and β-carotene content, while a modification of the lycopene isomeric composition was observed after heating. In detail, in the oil fraction of both the oleoresins, the heat treatments in the range from 60 °C to 100 °C induced a decrease of *cis*-isoforms of lycopene and an increase of all-*trans*-lycopene. This shift of the lycopene isomeric composition is similar in the two oleoresins and occurred at significant extent between 60 and 80 °C (Figure 6A,B,E,F). As a result a decrease of the ratio *cis*/*trans*-lycopene related to the increasing of temperature was observed.

Thus, in both the oleoresins (oil fraction) we observed a temperature dependent increase in all-*trans*-lycopene, and a decrease in *cis*-lycopenes likely due to retro-isomerization *cis-trans*, indicating a more stable isomeric composition compared to other oleoresins produced by traditional organic solvent extraction. *Cis* to *trans* retro-isomerization, following heat-treatment of tomato oleoresins and increase in *trans*-isomers by heating tomato sauces were reported [14].

In the two oleoresins, the lycopene measured in the insoluble fraction showed a quite opposite response to heat treatments (Figure 6C,D,G,H). In the oleoresin S-CO_2_ExT, *trans* and *cis*-isoforms of lycopene tend to decrease from 60 °C to 100 °C, compared to the sample kept at 25 °C. When the data were considered as percentage of the total carotenoids, it was evident that the ratio *cis*/*trans*-lycopene increased with the increasing of temperature, the crucial temperature being 80 °C (Figure 6D). This seems to confirm that, in S-CO_2_ExT, heating induced a degradation of the lycopene, probably through the *trans* to *cis* isomerization.

A different behavior during the heating was evident in the insoluble fraction of the oleoresin with added hazelnuts, ExCO_2_TH: an increase of both, *cis* and *trans*-isomers of lycopene, was observed after 60 °C and 80 °C treatments, compared to the sample at 25 °C. The further temperature increase at 100 °C induced a dropping of the *trans*-lycopene and a significant increase of the *cis*-isoforms (Figure 6G,H). This resulted in a significant change of the ratio *cis*/*trans* occurring at 100 °C confirming that the temperature of 100 °C is critical for the shift *cis* to *trans* lycopene isomerization.

Accordingly, the stability of lycopene-based products depends on their lycopene isomer profile in a complex manner. Some lycopene isomers are not stable and prone to retro-isomerization, according to the literature, 5-*cis* is the most stable among the predominant lycopene isomers followed by the all-*trans*, the 9-*cis* and the 13-*cis*. Besides, the composition of the source matrices, the temperature range and the treatment time can affect the stability of lycopene.

On the other hand, thermal isomerization of lycopene is known to improve its bioavailability from food matrices. However, to our knowledge, the bioavailability of individual lycopene isomers has not been yet investigated. It can be assumed that, as for lycopene stability, bioavailability of lycopene-based products is dependent on their lycopene isomer profile. Nevertheless, in our opinion, the assumption that only the technological processing affecting the isomeric profile can modulate the stability and bioavailability of lycopene-based products [14] is not obvious. The extreme variability in the physicochemical setting of the different varieties of oleoresins, with particular attention to the lipid environment, requires specific studies for each specific product and each processing procedure.

### 3.6. Effect of Temperature on Antioxidant Activity of Oleoresins

**Figure 7:**
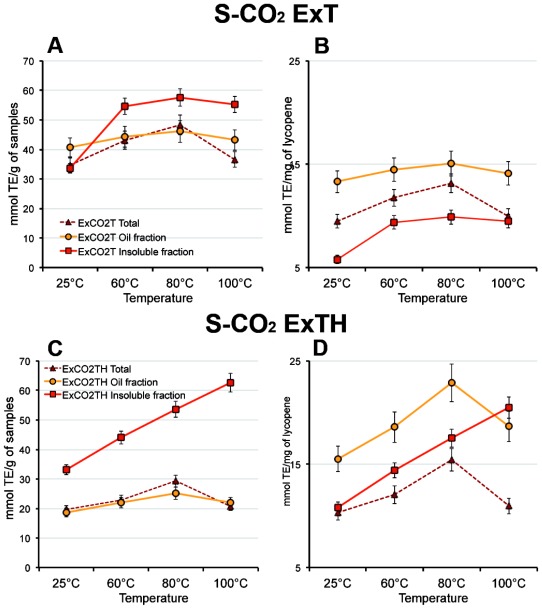
Effect of the temperature on antioxidant activity in the total extracts, oil- and insoluble-fractions of the S-CO_2_ extracted oleoresins. Antioxidant activity was expressed as mmol of trolox equivalent (TE) per gram of sample (**A**,**C**) or per mg of contained lycopene (**B**,**D**). S-CO_2_ExT: oleoresin extracted by S-CO_2_ from pure tomato powder; S-CO_2_ExTH: oleoresin extracted by S-CO_2_ from tomato powder added with hazelnut powder. Data are mean ± SD of three measures from three independent samples.

Figure 7 shows the effect of the heat-treatments on the antioxidant activity of the two oleoresins, ExCO_2_T and ExCO_2_TH as whole (Total), and as oil- and insoluble-fractions. As overall trend, it was observed that the antioxidant activity of both the oleoresins increased with the increase of the temperature, whereas the solvent extract lost almost completely its antioxidant activity at temperature above 60 °C (data not shown). In detail, the oil fraction of the oleoresins S-CO_2_ExT showed no significant changes of its antioxidant activity when submitted to heat treatments (60 °C, 80 °C and 100 °C) compared to the sample at 25 °C. Instead, the insoluble fraction increased significantly its antioxidant activity from 33 mmol TE/g of sample to a value around 55 mmol TE/g after heating at 60 °C, 80 °C and 100 °C with no significant differences among the three temperatures (Figure 7A). This result has been confirmed by referring the antioxidant activity values per mg of contained lycopene (Figure 7B). The whole oleoresin and oil fraction of the S-CO_2_ExTH (with hazelnut components), showed an increase of the antioxidant ability after the treatment at 80 °C, as compared to the 25 °C treated samples, and a dropping at 100 °C. This was confirmed by evaluating the antioxidant activity per mg of contained lycopene. Interestingly, the insoluble fraction of S-CO_2_ExTH oleoresin displayed a linear rising of antioxidant ability as the temperature increased. The 100 °C treatment induced the doubling of the antioxidant value (from about 33 to 63 mmol TE/g of oleoresin) (Figure 7C). These results are independent from the increase of lycopene content after heating (Figure 7B) as confirmed when the antioxidant activity was evaluated per mg of lycopene, (Figure 7D). It can be hypothesized that in this oleoresin, thermal treatment increased solubility of lycopene, which is in a physicochemical environment making it also a more efficient antioxidant.

## 4. Conclusions

This study presents data on thermal stability and antioxidant activity of oleoresins extracted by S-CO_2_ methodology, and on the analysis of the geometrical isomers of lycopene. The considered oleoresins, S-CO_2_ExT and S-CO_2_ExTH, showed a peculiar bi-phasic oil-solid appearance, and an unique isomeric composition, with higher *cis*-lycopene content, enhanced antioxidant ability and thermal performances, compared with traditional solvent extracts. These results indicated a putatively more bioavailable lycopene in the S-CO_2_ extracted oleoresins than in tomato-based foods, because of the lycopene isomeric composition more similar to that found in the human serum. The complexity of behavior of chemical species in different physicochemical environments could also explain the contradictory results of the epidemiologic and biochemical studies where the lycopene source was different (pure compounds, extracts, oleoresins or tomato-based foods). In addition, our results on the biological activity on human cell cultures of S-CO_2_ extracted oleoresins with hazelnut [7] or grape seeds [5] added to tomato matrix, demonstrated the higher bioactivity of these oleoresins compared with organic solvent extracts. Therefore, it becomes paramount to determine the geometrical configurations of major carotenoids during analyses that pertain the putative biological activity, mainly when studies on human cell cultures are performed. Information gained from this study, together with reports on biological isomer levels, allows the further consideration of the biological relevance of lycopene isomers. To date, the most commercially available lycopene extracts exhibit isomeric profiles quite similar to the starting tomato sources, whether they are derivatives or extracted by organic solvent. S-CO_2_ extraction, especially in presence of oleaginous seed added matrix, offers a one-step process for lycopene extraction, solubilization and formulation. Finally, the presence of potentially allergenic hazelnut proteins in the oleoresins [21] requires careful consideration. Information gained from this study will we enable to estimate of the healthy potential of processed products, obtained by innovative extraction technologies, enriched in some key antioxidants and especially in specific geometrical isomers.

## Figures and Tables

**Table 1:** Quali-quantitative analysis of carotenoids contained in the oil- and insoluble-fraction of the two S-CO_2_-extracted oleoresins from pure tomato powder (S-CO_2_ExT) and from tomato/hazelnut powder (S-CO_2_ExTH). Values are expressed as μg/g of oleoresin and as percentage of the total carotenoids. Data are mean ± SD of three measures from three independent samples.

*Carotenoids*	S-CO_2_ Oleoresins								Solvent extract

	S-CO_2_ ExT a				S-CO_2_ ExTH b				Tomato matrix

	Oil fraction		Insoluble fraction		Oil fraction		Insoluble fraction		

	μg/g of oleoresin	% of carotenoids	μg/g of oleoresin	% of carotenoids	μg/g of oleoresin	% of carotenoids	μg/g of oleoresin	% of carotenoids	% of carotenoids
Lutein	193 (±12)	4.8	149 (±9)	2.3	34 (±7)	2.53	50 (±5)	1.5	0.9
β-carotene	801 (±22)	19.8	549 (±19)	8.4	145 (±17)	10.7	99 (±7)	3.1	4.7
*cis*-lycopene	2353 (±88)	58	1539 (±78)	23.6	782 (±34)	57.77	385 (±10)	12	9.9
*trans*-lycopene	706 (±18)	17.4	4277 (±120)	65.7	392 (±10)	28.99	2677 (±71)	83.4	84.5
Total lycopene	3060	75.5	5816	89.3	1175	86.77	3062	95.4	94.4
Total carotenoids	4056	100	6515	100	1354	100	3211	100	100
Lycopene (mg/100 g of oleoresin)	306		581.6		117.5		306.2		119

a Oleoresin extracted by supercritical CO_2_ from tomato matrix;

b Oleoresin extracted by supercritical CO_2_ from tomato/hazelnut matrix.

**Table 2:** Percentage of fatty acids of lipid fraction extracted from dried tomato powder and supercritical CO_2_ extracted oleoresins, from tomato and tomato/hazelnut matrices. Values are expressed as percentage of the total lipids. Data are mean ± SD of four measures from three independent samples.

Fatty acids	Tomato powder	S-CO_2_-Extracted-Oleoresins	

		S-CO_2_ ExT a	S-CO_2_ ExTH b
*Percentage* (*%*)			
Palmitic acid (16:0)	23.77 (*1.88*)	5.59 (*0.32*)	5.24 (*0.22*)
Palmitoleic acid (16:1)	n.d. c	n.d. c	0.12 (*0.01*)
Stearic acid (18:0)	2.98 (*0.05*)	2.61 (*0.05*)	2.55 (*0.04*)
Oleic acid (18:1)	4.22 (*0.98*)	80.46 (*2.59*)	83.04 (*3.03*)
Linoleic acid (18:2)	52.59 (*1.78*)	9.33 (*0.77*)	8.9 (*0.81*)
Linolenic acid (18:3)	7.38 (*0.57*)	0.71 (*0.03*)	0.15 (*0.01*)
Others d	9.06 (*0.2*)	1.3 (*0.01*)	n.d. c

a Oleoresin extracted by supercritical CO_2_ from tomato matrix;

b Oleoresin extracted by supercritical CO_2_ from tomato/hazelnut matrix;

c not detected;

d unidentified compounds.

## Abbreviations

S-CO_2_: supercritical carbon dioxide
S-CO_2_ExT: oleoresin extracted from pure tomato powder
S-CO_2_ExTH: oleoresin extracted from tomato powder added with an equal amount of dry hazelnut powder
TE: Trolox equivalents
FA: fatty acid
PUFA: polyunsaturated fatty acid
